# Nano/microvehicles for efficient delivery and (bio)sensing at the cellular level

**DOI:** 10.1039/c7sc02434g

**Published:** 2017-08-21

**Authors:** S. Campuzano, B. Esteban-Fernández de Ávila, P. Yáñez-Sedeño, J. M. Pingarrón, J. Wang

**Affiliations:** a Department of Analytical Chemistry , Complutense University of Madrid , E-28040 Madrid , Spain . Email: susanacr@quim.ucm.es ; Email: pingarro@quim.ucm.es; b Department of Nanoengineering , University of California , La Jolla , San Diego , California 92093 , USA . Email: josephwang@ucsd.edu; c IMDEA Nanoscience , Ciudad Universitaria de Cantoblanco , 28049 Madrid , Spain

## Abstract

A perspective review of recent strategies involving the use of nano/microvehicles to address the key challenges associated with delivery and (bio)sensing at the cellular level is presented. The main types and characteristics of the different nano/microvehicles used for these cellular applications are discussed, including fabrication pathways, propulsion (catalytic, magnetic, acoustic or biological) and navigation strategies, and relevant parameters affecting their propulsion performance and sensing and delivery capabilities. Thereafter, selected applications are critically discussed. An emphasis is made on enhancing the extra- and intra-cellular biosensing capabilities, fast cell internalization, rapid inter- or intra-cellular movement, efficient payload delivery and targeted on-demand controlled release in order to greatly improve the monitoring and modulation of cellular processes. A critical discussion of selected breakthrough applications illustrates how these smart multifunctional nano/microdevices operate as nano/microcarriers and sensors at the intra- and extra-cellular levels. These advances allow both the real-time biosensing of relevant targets and processes even at a single cell level, and the delivery of different cargoes (drugs, functional proteins, oligonucleotides and cells) for therapeutics, gene silencing/transfection and assisted fertilization, while overcoming challenges faced by current affinity biosensors and delivery vehicles. Key challenges for the future and the envisioned opportunities and future perspectives of this remarkably exciting field are discussed.

## Abstract 2

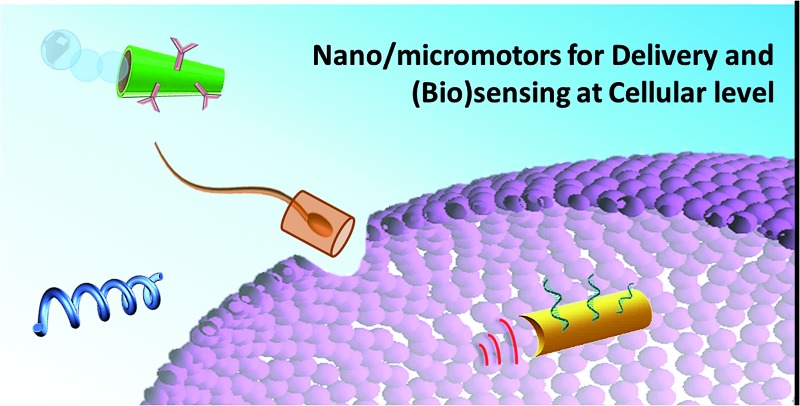
A perspective review of recent strategies involving the use of nano/microvehicles to address the key challenges associated with delivery and (bio)sensing at the cellular level is presented.

## Introduction

The ability to probe cellular activity and target therapeutics inside living cells is of considerable importance as we strive to develop tiny biosensors that monitor complex cellular events and design therapies that can modulate the processes that occur within cells. Such intracellular delivery of drugs can sharply increase the efficiency of various treatment protocols. Nanotechnology researchers have thus been aiming at developing novel nanovehicles capable of entering target cells, probing intracellular processes and ferrying their payload to sub-cellular organelles. Despite the considerable progress made in recent years in biosensing and delivery at the cellular level, current systems are still not able to address some important challenges, such as achieving ultrasensitive detection in a short time in microscale environments, and releasing functional cargoes in a quick and controlled way at specific extra- and intra-cellular locations with limited accessibility. The broad scope of operations and applications, along with the ultrasmall dimensions, accessibility and force offered by synthetic nano/micromotors, open up intriguing possibilities for these purposes, overcoming some of the unfilled gaps and unmet challenges. In this field, apart from chemically powered nano/micromotors propelled by toxic (*e.g.* H_2_O_2_) or biofriendly (*e.g.* glucose, urea) fuels, fuel-free nano/micromotors, powered by various external stimuli based on magnetic, electric or ultrasonic fields, exhibit major advantages in directional motion control and have long lifetimes and excellent biocompatibility.

A tremendous effort has been made over the past decade for the implementation of strategies to design and fabricate nano/micromotors with different functionalities. These artificial nano/micromachines can be designed to accomplish challenging biomedical applications, such as biosensing and targeted drug, gene, protein and cell delivery, both at the extra- and intra-cellular levels. However, several critical challenges should be more deeply addressed, such as improving biocompatibility through smart functionalization, achieving efficient propulsion in complex biological media such as intravascular fluids, achieving safe removal from the body once the mission has finished (involving the use of self-degrading nanomotors fabricated with biodegradable transient materials) and enhancing the cooperation of multiple nano/micromotors for complex tasks.

Although these unsolved critical issues should be addressed, the high performance demonstrated by nano/micromotors for biosensing and targeted payload delivery at the cellular level has encouraged the scientific community to devote more effort to understanding the fundamental science behind nano/micromotors and expanding their applications. Further developments are expected to provide intelligent, responsive and multifunctional nano/micromachines that mimic the amazing functions of natural systems for a diverse range of biomedical applications. These will allow unprecedented levels of cell manipulation and targeted intervention even at the single cell level, with the possibility of treating diseases more specifically and safely. The immense progress and benefits that nano/micromotors can bring to the fields of biosensing and delivery at the cellular level, along with the potential challenges that lie ahead and the existing gaps and limitations, are discussed in the following sections.

## Nano/microvehicles

Nano/microvehicles are expected to become powerful mobile biosensors^1,2^ and active transport systems, holding great potential for a variety of diagnostic and therapeutic applications. The design of miniaturized and versatile nano/microvehicles would allow access throughout the whole human body, leading to new procedures down to the cellular level, and offering localized diagnosis and treatment with greater precision and efficiency.^3,4^ Such nano/micromotors with medical potential must be fabricated with non-toxic materials and optimized shapes, and must be propelled by powerful and biocompatible propulsion mechanisms.

A wide range of nano/microvehicles based on different propulsion mechanisms have been developed in recent years.^5,6^ These vehicles can be classified into three different types based on their propulsion mechanism: (1) those with their own built-in propulsion powered by energy-rich molecules, (2) those with external energy sources such as magnetic or acoustic fields, and (3) systems “towed” to the site of interest by living objects with propulsion functions. In the case of autonomous systems, the engine must be equipped with some sort of navigation system to direct the nano/microvehicle to the target, while the direction of externally powered systems must be guided by an operator.^6^ Another widely accepted classification divides nano/microvehicles into only two major groups: (1) those that are chemically propelled^5^ and (2) fuel-free nano/micromotors powered by biocompatible external energy sources, such as magnetism and ultrasound (US) (and sometimes optical, thermal and electrical energy).^4,7–9^ Catalytic nano/micromachines include nano/micromotors powered by external^2,10–15^ or natural^16,17^ fuels, and micromotors with chemotaxis behavior towards a particular substrate.^18,19^ Magnetically actuated flexible/helical swimmers,^20–25^ US-powered nanomotors^26–30^ and self-motile cell-based micromotors are included in the fuel-free group.^31,32^ Many of these nano/micromotors show considerable promise for navigation through complex biological fluids, reaching hard-to-access bodily locations, or releasing their therapeutic payloads at predetermined body destinations.

Fuel-propelled micromotors require a specific fuel and specific composition of their surface. They can drive themselves through aqueous solution using surface reactions to generate local gradients of concentration, electrical potential and gas bubbles.^33^ Among the chemically powered nano/micromotors, tubular microengines are particularly attractive for biosensing applications due to their efficient bubble-induced propulsion resulting from the catalytic decomposition of fuel on their internal surface. Fuel-propelled nano/micromotors can perform different types of motion, and all of them are limited by access to fuel in the medium. The inherent lack of a defined trajectory can be solved by including a magnetic component (*e.g.* nickel) in their structure, thus allowing their guidance in the presence of an external magnetic field.^34^

The development of biocompatible and biodegradable nano/micromotors, driven by natural fuel sources inherently created in biological systems, such as glucose, urea,^35–38^ acid^16,17^ or water,^39–43^ is crucial for *in vivo* applications^17,44^ because these systems not only circumvent the need for an external fuel, but also provide selective nano/micromotor activation only in particular environments.

Wang’s group has reported the use of body fuel-powered micromotors, such as Zn or Mg-based micromotors, for *in vivo* drug delivery applications. Indeed, this group reported the first example of the use of micromotors *in vivo*, showing the propulsion of Zn motors in the harsh acidic environment of a mouse’s stomach.^16^ Mg micromotors coated with a pH-responsive enteric polymer were also demonstrated to be useful for site-specific gastrointestinal (GI) delivery.^45^ These micromotors safely passed through the gastric fluid and were selectively activated in the GI tract of living mice, demonstrating important advantages toward localized tissue delivery. Mg-based micromotors have also been combined with a cargo-loaded pH-responsive film, demonstrating that they can autonomously and temporally neutralize gastric acid, triggering a responsive payload release in the mouse’s stomach.^46^ This simple but efficient motor configuration opens up future prospects in drug delivery, offering a unique rational on-board release in the presence of specific microenvironments.

Externally-powered motors, using mainly magnetic and US energies to drive their motion, are extremely interesting for *in vivo* biomedical applications due to their potential biocompatibility and independence of the chemical composition of the medium in which they operate. The use of magnetic fields to power nano/microswimmers has gained particular interest as magnetic fields can penetrate the human body, allowing for the wireless control of these tiny devices without harming cells and tissues.^23^ A special type of magnetic helical microswimmer is artificial bacterial flagella (ABF), which can perform 3D navigation in a controllable fashion with micrometer precision in liquid, using low-strength rotating magnetic fields (1000 times lower than the fields used in MRI systems), translating rotational movement into translational motion in a screw-like manner.^23,47^ Moreover, Felfoul *et al.* ^48^ demonstrated that harnessing swarms of microorganisms exhibiting magneto-aerotactic behavior can significantly improve the delivery efficiency of drug nanocarriers to tumor hypoxic regions of limited accessibility.

Nanomotors propelled by acoustic forces also offer great advantages in terms of biocompatibility and cargo delivery. It has been demonstrated that US propulsion facilitates the internalization of functionalized nanowire (NW) motors into living cells and enables them to remain acoustically active, undergoing both directional motion and spinning inside the cells.^29,49^ The pioneering work of Mallouk and colleagues at Penn State University demonstrated for the first time that such a gold NW motor, powered by ultrasonic waves, was able to move inside HeLa cells without causing any damage.^49^ The introduction of US-powered NW motors into living cells has opened a new door for payload delivery and (bio)sensing at the cellular level. In addition, US actuation opens up the possibility of driving and controlling nano/micromotors using deeply penetrative (yet medically safe) US to create powerful micromotors with remarkable tissue penetration properties that will be discussed in the following sections.^27,28^

The use of self-motile cell-based micromotors provides great flexibility regarding the type of flagellated organism that can be integrated into the microbiomotor. Moreover, the motile cells employed do not need to be cultivated and are readily available, easy to handle, able to swim in highly viscous media and completely harmless.^50^ Motile cells have been used as microbiomotors in connection with metal-coated polymer microhelices^31^ or magnetic microtubes.^32,50^

These tiny devices can also be classified according to their shape, size and material building block, which determine their propulsion mechanism and the best fabrication route for their preparation.^51^ Different metal NWs, the simplest 1D structure used as nano- and micromotors, are fabricated by metal electroplating inside a membrane template (usually an anodic aluminum oxide or polycarbonate membrane). These commercially available membranes are sputtered with gold to act as a working electrode in the fabrication process, and are dissolved in the last step using acid or base solutions to release the as-prepared NWs. Janus spherical nano/micromotors, consisting of two hemispheres playing different roles (one relating to the propulsion ability, and the other relating to targeted application), are commonly fabricated by catalytic metallic (*e.g.* Ir, Au, Pt or Ti) thin film deposition (by electron beam evaporation, atomic layer deposition or sputtering processes) or polymeric coating onto the surface of nano- or microbeads of different materials (*e.g.* SiO_2_, polystyrene or Mg) uniformly distributed on glass slides or silicon wafers. Tubular nano/micromotors are different to those using NWs as they have tubular structures with specific inner and outer parts, both of which are suitable to be functionalized.^24^ Early tubular micromotors (microtubes, microrockets or microjets) were fabricated by a rolling-up process based on the patterning of a photoresist on a substrate (*e.g.* Si wafer) bearing a sacrificial layer.^33^ The subsequent evaporation of metals at a glanced angle created a window that determined the rolling process (occurring after etching the substrate). However, smaller sized microtubes, which are more compatible with certain applications, can be fabricated in a simplified manner by template electroplating using appropriate membranes with conical shaped pores.^52^ This methodology avoids the need for a cleanroom and significantly reduces the cost and fabrication time. It is also worth mentioning that various chemical compounds, such as polyaniline (PANI), poly(3,4-ethylenedioxythiophene) (PEDOT), polypyrrole (PPy), poly(3-aminophenylboronic acid) (PAPBA) and molecularly imprinted polymers (MIPs), can be electrodeposited allowing facile further modification. This is therefore an attractive route for fabricating versatile microtubes to be functionalized for different applications. Interestingly, all of these fabrication routes offer many alternative ways to tailor the size, shape, propulsion mechanism and final properties of the synthesized nano/micromotors, as desired for achieving their optimum performance in the pursued applications.

## Nano/microvehicles for biosensing and delivery

Affinity biosensors involving analyte–receptor interactions under static conditions have been demonstrated to achieve low detection limits. However, ultrasensitive detection in a short time remains a major challenge due to the small sample volumes and difficulty enhancing analyte–receptor interactions through enhanced mass transport in such microliter samples. The functionalities and capabilities exhibited by advanced nano/micromotors have opened the way to new biosensing applications in previously inaccessible microscale environments. The easy functionalization of the nano/micromotor structure with specific biological or artificial recognition elements, attached onto the motor surface or embedded in the motor material itself, has allowed for their implementation as dynamic biosensing systems, offering specific and fast detection of different target analytes, greatly accelerated by the efficient and fast motion of receptor-functionalized nano/micromotors through the sample, without or with minimal sample treatment.^53,54^

These novel motile biosensors, in which the continuous movement of receptor-functionalized nano/micromotors around the sample increases the likelihood of target–receptor contacts, have been demonstrated to be highly selective and sensitive, eliminating time-consuming washing steps and largely simplifying and accelerating the overall biosensing procedure. In the particular case of fuel-propelled tubular microrockets, the fast motion and bubble release trigger local vortexes that promote the transport of the target analyte toward the functionalized microjet outer surface, offering the possibility to perform rapid “on-the-fly” recognition events.^51,55^

On the other hand, the development of a new generation of drug delivery vehicles capable of encapsulating, transporting and releasing active substances in a rapid, targeted and controlled manner is of great interest. One of the biggest challenges to face in targeted drug delivery is conquering the innate immune system of the host organism, which readily recognizes and destroys foreign material that enters the body’s circulation.^6,56^ Over the past decade, many nanomaterial-based systems have been developed for drug delivery applications, including liposomes, polymeric nanoparticles, dendrimers, polymersomes and nanoemulsions.^57^ However, despite the significant progress made to improve the therapeutic efficacy of such drug delivery nanosystems, important challenges, such as issues relating to off-targeting, compatibility with the immune system, low cell and tissue penetration and low therapeutic efficacy, have not yet been overcome. This demands the development of innovative technologies.

In this context, recent delivery strategies involving the use of nano/micromotors have demonstrated the integration of self-driven and navigation capabilities, achieving not only smart drug encapsulation and release but also precise guidance and control.^13^ It is important to mention at this point (as will be described in more detail later) that the clever delivery approaches proposed by Schmidt’s group, using artificially motorized sperm cells^31^ and sperm-hybrid micromotors,^32^ have demonstrated the ability to inhibit the immune response using the specific glycans on the sperm cell membrane. Although still at an early stage, these recent advances will play a major role in the future design of new micro/nanodevices with low immunogenicity that are able to bypass the immune system.

Nano/micromotors possess a high towing force that represents a major advantage for their implementation as cargo delivery platforms. Furthermore, their directionality can be controlled by the incorporation of magnetic materials in their structure, allowing for the precise motion control required for targeted drug delivery.^34^ In addition, the easy functionalization of the nano/micromotor structure with specific bioreceptors has also enabled efficient modification with a great variety of cargoes and their delivery, both in extra- and intra-cellular spaces. Therefore, drug delivery nano/microvehicles promise to address several key issues for the development of more powerful therapeutics, including the evasion of immune-mediated clearance, improvements to therapeutic efficacy, enabling the combinatorial delivery of multi-cargoes, avoiding negative side effects by delivering the drug precisely at the disease-specific place, and overcoming limited access to certain areas of the body with low drug tissue distribution and cellular barrier penetration.^58,59^

**Fig. 1:**
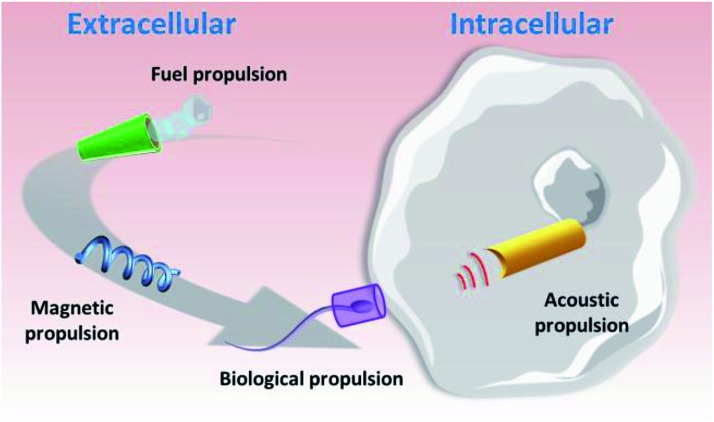
The main types of nano/micromotor and their propulsion mechanisms (catalytic, magnetic, biological and acoustic propulsion) for biosensing and delivery at the extra- and intra-cellular levels.

Taking into account the breakthrough biomedical applications of nano/micromotors demonstrated by pioneering groups in this field^4^ and the compatibility of operation demonstrated in cellular media,^51^ we journey in this perspective article through recent advances in the use of nano/microvehicles for cellular biosensing and cargo delivery applications. Selected examples of such motor-based operations will be discussed, along with related challenges, envisioned opportunities and future perspectives. As will be discussed below, nano/micromotors have been applied for biosensing and delivery both in extra- and intra-cellular spaces. As depicted in Fig. 1, while catalytic, magnetic and motile cell-propelled vehicles have been preferably used for extracellular applications, US-propelled nanomotors are the preferred choice for intracellular operations.

## Nano/microvehicles for biosensing at the cellular level

The ability to dynamically probe intracellular processes and monitor key molecules in individual cells is the next frontier in biomedicine. As introduced above, the nano/micromotor surface can be functionalized with specific bioreceptors used for the detection and/or isolation of different biological targets under dynamic conditions at the extra- and intra-cellular levels. The continuous movement of synthetic nano/micromotors through the samples, and the dramatic modulation of mass transport by the vortex effect resulting from the locomotion of bubble-propelled microengines, significantly enhance the interactions of the nano/micromotor sensing surfaces with the target analytes, thereby improving the recognition efficiency and offering ‘on-the-fly’ recognition of specific biomolecular interactions.^51,60^ The different biosensing strategies based on receptor-functionalized nano/micromotors are summarized in Table 1.

**Table 1:** Nano/microvehicles developed for biosensing at the cellular level

Type	Propulsion	(Bio)receptor	Target analyte	Target cells	Average speed^*a*^, μm s^–1^	Ref.
Ti/Fe/Au/Pt microtubes	H_2_O_2_	Anti-CEA monoclonal antibody	CEA on the cell surface	BxPC-3	85	11
Au/Ni/PANI/Pt microtubes	H_2_O_2_	Lectin (ConA)	Specific terminal carbohydrates of the bacterial cell surface	*E. coli*	80	12
PAPBA/Ni/Pt microtubes	H_2_O_2_	PAPBA	Sugar residues on yeast cell walls	Yeast cells	80	61
Millimeter-sized tubular motors	H_2_O_2_	HRP	H_2_O_2_ released in extracellular space	Tumoral kidney tubular cells	—	2
GO-coated AuNWs	Acoustic	Dye-labeled ssDNA	Intracellular miRNA-21	MCF-7	—	62

^*a*^Value dependent on the fuel concentration and the strength of the magnetic and UV fields. AuNWs: Au nanowires; ConA: concanavalin A; CEA: carcinoembryonic antigen; GO: graphene-oxide; HRP: horseradish peroxidase; PAPBA: poly(3-aminophenylboronic acid); PANI: polyaniline; ssDNA: single-stranded DNA.

**Fig. 2:**
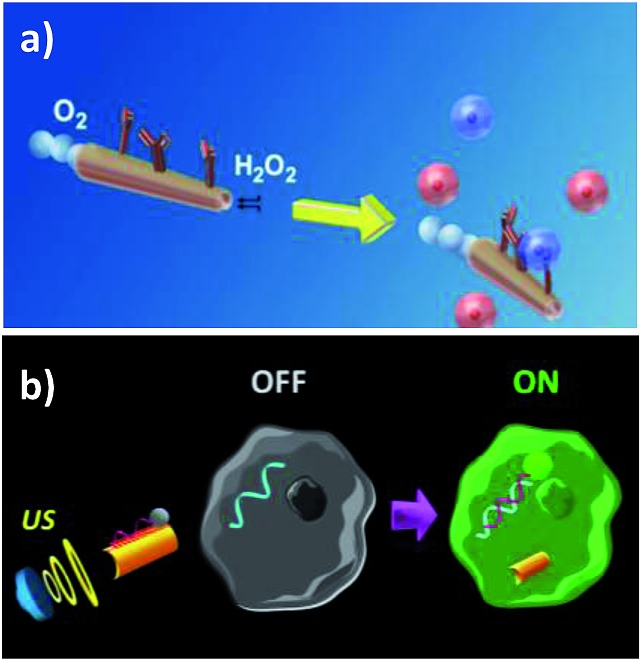
Nano/micromotors for extra- (a) and intra- (b) cellular biosensing. (a) Anti-CEA-modified microrockets for the capture and isolation of cancer cells. (b) Intracellular detection of miRNA-21 by US-propelled ssDNA@GO-functionalized nanomotors. Reprinted from ref. 11 (a) and ref. 62 (b) with permission.

The developed methodologies were designed for the specific detection of relevant targets both at extra- (proteins, antigens and carbohydrates of cellular surfaces, and H_2_O_2_ release to extracellular media) and intra- (mature miRNAs) cellular levels. For example, Balasubramanian *et al.* ^11^ described an immuno-micromachine constructed by anti-carcinoembryonic antigen (CEA) monoclonal antibody modification of Ti/Fe/Au/Pt microrockets prepared by standard photolithography. The modified microvehicle was employed for the *in vitro* isolation and transport of cancer cells by means of the selective biosensing of CEA overexpressed in pancreatic cancer cells (Fig. 2a). The micromotors showed sufficient propulsive force for the efficient transport of the captured target cells even in complex biological media. The same group also reported attractive strategies to isolate bacterial and yeast cells using lectin-modified or boronic acid-based microengines with small sizes, mass produced through a low-cost membrane template electrodeposition technique.^12,61^ These concepts were demonstrated for the rapid and real-time isolation of *Escherichia coli* using Au/Ni/PANI/Pt microtubular engines functionalized with concanavalin A and yeast cells using PAPBA/Ni/Pt microtube engines. The multifunctional capabilities of the concanavalin A-modified Au/Ni/PANI/Pt microtubular engines were shown by performing the capture and transportation of polymeric drug carrier particles for theranostic purposes and the triggered release of the captured cargoes.

Moreno-Guzmán *et al.* ^2^ described the use of millimeter-sized tubular motors for the electrochemical and optical sensing of H_2_O_2_ in conjunction with 3,3′,5,5′-tetramethylbenzidine (TMB) in just 120 s. This concept, which relied on the asymmetric and continuous release of SDS surfactant and fresh horseradish peroxidase (HRP) from the tubular motors into the sample solution, was applied for the determination of H_2_O_2_ in cultures of tumor kidney tubular cells.

All of these studies point out the great advantages offered by catalytic nano/micromotors in terms of their faster sensing capacity also at the cellular level. However, the major challenge to face for their potential *in vivo* application is still their need of H_2_O_2_ fuel for propulsion. With this in mind, motors propelled by external stimulation, such as acoustic fields, appear to be great candidates to work in cell culture environments. The strong energy of the acoustic fields can be utilized to internalize and move nanomotors inside living cells,^49^ and therefore the efficient movement of US-powered nanomotors can be exploited for intracellular sensing. An illustrative example of this strategy is the construction of a single-step nanomotor for rapid intracellular miRNA biosensing at the single cell level.^62^ The concept relied on the use of US-propelled dye-labeled single-stranded DNA (ssDNA)/graphene-oxide (GO)-coated gold NWs (AuNWs), with fluorescence quenched by the π–π interaction between GO and the dye-labeled ssDNA. After cell internalization, and in the presence of the target miRNA, the fluorescence signal is recovered due to the displacement of the dye-ssDNA probe from the GO-quenching motor surface, leading to attractive intracellular “OFF–ON” fluorescence switching (see Fig. 2b). The ability of the US-powered ssDNA@GO-functionalized nanomotors to screen cancer cells was demonstrated by measuring in a few minutes the endogenous content of target miRNA-21 in two types of intact cancer cell (MCF-7 and HeLa) with significantly different expression levels of miRNA-21. The fast cell internalization process of the nanomotors and their rapid intracellular movement under the acoustic field led to major improvements in the sensitivity and sensing speed. The results presented demonstrated that while ∼60% recovery of the fluorescence intensity was observed within 5 min when applying US at 9 V, 30 min were required to recover half of the fluorescence under static conditions.

## Nano/microvehicles for delivery at the cellular level

Nano/microvehicles have demonstrated considerable promise for the delivery of a wide variety of cargoes (drugs, nano- and magnetic particles, cells, oligonucleotides and proteins), both in intra- and extra-cellular spaces. Table 2 summarizes the relevant strategies for cargo delivery at the cellular level using nano/microvehicles. The methods are discussed in the following subsections by classifying them in accordance with the type of delivered cargo.

**Table 2:** Nano/microvehicles developed for cargo delivery at the cellular level

Type	Propulsion	Cargo	Target cells	Average speed^*a*^, μm s^–1^	Ref.
Protein-functionalized AuNWs	Electrical	NF-kB + TNFα	HeLa	≤50	63
Janus MSNs	H_2_O_2_	Drug (DOX)	HeLa	20.2	14
Catalase-modified Janus capsules	H_2_O_2_	Drug (DOX)	HeLa	232	13
Biodegradable self-propelled PLL/BSA-multilayer rockets	H_2_O_2_	Drug (DOX)	HeLa	68	15
Polymer stomatocytes functionalized with PtNPs	Chemotaxis towards H_2_O_2_	Drug (DOX)	Neutrophils	—	18
PEG-*b*-PCL and PEG-*b*-PS-hybrid-based stomatocytes	Chemotaxis towards H_2_O_2_	Drug (DOX)	HeLa	39	19
PEDOT/Zn microtubes	Acid-powered	AuNPs	Stomach cells	—	45
Flexible nanoswimmers	Magnetic	Drug (DOX)-loaded microparticles	HeLa	10	21
ABFs	Magnetic	Drug (calcein)	C2C12 mouse myoblasts	8.4	22
Liposome functionalized-ABFs	Magnetic	pDNA	HEK 293	43.9	23
Multifunctional nanotubes	Magnetic	MB	MDA-MB-231	—	24
Dual-action biogenic hybrid micromotors	Magnetic	Camptothecin	HeLa	—	25
Magneto-aerotactic microorganisms (MC-1)	Magnetic	Drug (SN-38)-loaded nanoliposomes	Tumor hypoxic regions	—	48
Hybrid magnetoelectric nanomotors	Magnetic	Drug (paclitaxel)	MDA-MB-231	—	64
Highly porous AuNWs	Acoustic	Drug (DOX)	HeLa	60	26
Nanoshells	Acoustic	3.3 μm magnetic beads	MCF-7	1050	28
AuNWs	Acoustic	siRNA	HEK-293-GFP and MCF-7-GFP	—	29
AuNWs	Acoustic	CASP3	AGS cells	37	30
Metal-coated polymer microhelices	Self-propelled	Sperm cells	Oocyte	19.7	31
Sperm-hybrid micromotors	Self-propelled	Drug (DOX)	HeLa cells and spheroids	—	32

^*a*^Value dependent on the fuel concentration and the strength of the magnetic and UV fields. ABF: artificial bacterial flagella; AuNWs: gold nanowires; BSA: bovine serum albumin; CASP-3: caspase-3; DOX: doxorubicin; MB: methylene blue; MC-1: the *Magnetococcus marinus* strain; MSNs: mesoporous silica nanomotors; NF-kB: nuclear factor-kappaB; PEDOT: poly(3,4-ethylenedioxythiophene); PLL: poly-l-lysine; pDNA: plasmid DNA.

### Drug delivery at the cellular level using nano/microvehicles

**Fig. 3:**
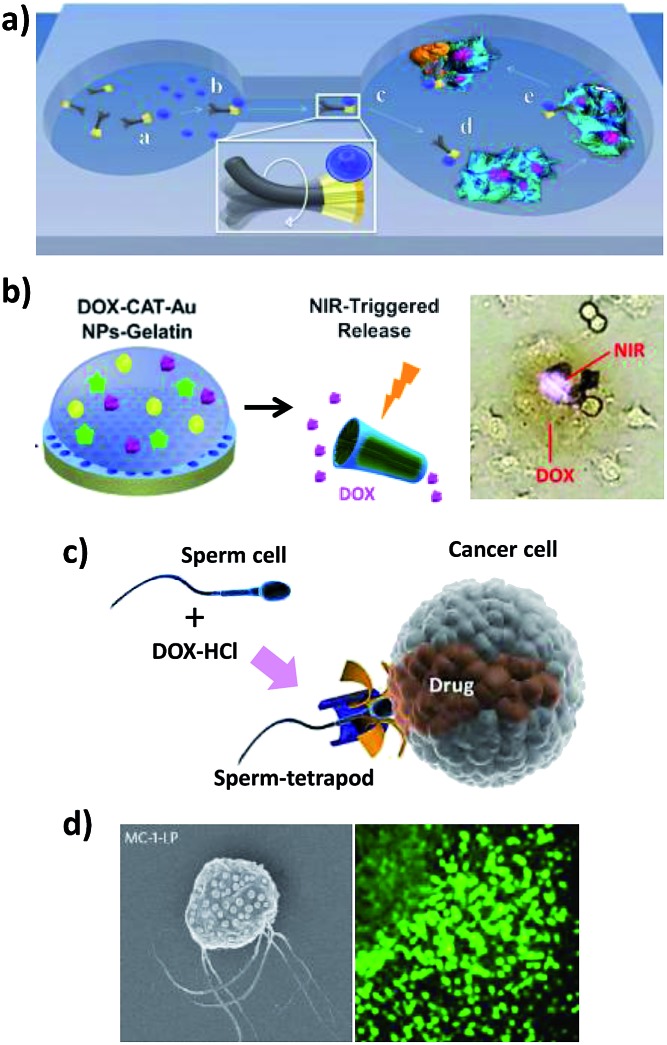
Different strategies for tumor and cell targeted drug delivery using nano/microvehicles. (a) Delivery of DOX-loaded magnetic polymeric particles using magnetically-driven flexible nanoswimmers. (b) Biodegradable (PLL/BSA)10-DOX-CAT-AuNPs-gelatin rockets for light-triggered DOX release. (c) Tumor targeted drug delivery using a sperm-hybrid micromotor under magnetic guidance with a mechanical sperm release trigger. (d) Magneto-aerotactic bacteria to deliver drug-containing nanoliposomes to tumor hypoxic regions. SEM of a *Magnetococcus marinus* strain (MC-1) cell with ∼70 SN-38 drug-loaded nanoliposomes attached. Reprinted from ref. 21 (a), ref. 15 (b), ref. 32 (c) and ref. 48 (d) with permission.

Nano/micromotors aim to deliver a drug selectively to the target tissues and cells with increased efficacy while reducing the side effects. The ability of nano/micromotors to transport therapeutic carriers to specific cellular locations such as cancer cells has been demonstrated by several groups. Wang’s group^21,65^ reported for the first time the directed delivery of magnetic polymeric particles loaded with the chemotherapeutic drug doxorubicin (DOX) using magnetically driven flexible nanoswimmers (Fig. 3a). These authors designed the fundamental mechanism of their fuel-free NW motors to have a cargo-towing ability, and they demonstrated the potential *in vitro* application using the directed delivery of drug-loaded microparticles at a speed of 10 μm s^–1^ through a microchannel from the pick-up zone to the release microwell containing HeLa cancer cells.

Mhanna *et al.* ^22^ reported the use of functionalized helical magnetic “microvoyagers” based on liposome functionalized artificial bacterial flagella (ABF) to perform *in vitro* single-cell-targeted drug delivery. The results presented demonstrated the feasibility of these microswimmers, steerable with an external magnetic field, to deliver the hydrophilic model drug calcein to C2C12 mouse myoblasts. The uptake of the drug by the target cell was monitored using fluorescence microscopy.

Wu *et al.* ^13,66^ proposed the use of self-propelled multilayer Janus capsule motors for effective drug transportation and controlled release at HeLa cancer cells. These hybrid biocatalytic Janus motors were based on hollow polyelectrolyte multilayer capsules and PtNPs^66^ or catalase,^13^ and were propelled by the biocatalytic decomposition of peroxide fuel. The encapsulated drug (DOX) could be rapidly released at predefined sites by US^66^ or NIR light^13^ triggering. The same group also reported the first example of self-propelled Janus mesoporous silica nanomotors (MSNs) with sub-100 nm diameters for drug encapsulation and intracellular delivery.^14^ These Janus nanomotors employed mesoporous silica nanoparticles with chromium/Pt metallic caps and were propelled by decomposing H_2_O_2_ to generate oxygen as a driving force with speeds of up to 20.2 mm s^–1^. The Janus MSNs were applied *in vitro* for intracellular localization and the slow release of the encapsulated drug (DOX) inside HeLa cells through the catalytic hydrolysis of lipid bilayers by cellular enzymes.

The DOX delivery and NIR light-controlled release capabilities at HeLa cancer cells were also described using US-powered highly porous AuNW motors, which exhibited a large surface area and a high drug loading capacity.^26^ Another approach involved the construction of biodegradable, self-propelled bovine serum albumin/poly-l-lysine (PLL/BSA) multilayer microrockets.^15^ The microrockets’ preparation involved a template-assisted layer-by-layer assembly of PLL/BSA layers followed by the incorporation of a heat-sensitive gelatin hydrogel, which greatly improved the encapsulation capacity, containing AuNPs, DOX and catalase (Fig. 3b). Interestingly, these protein-based microtubes can be enzymatically degraded under physiological conditions after completing their missions.

Xu *et al.* ^32^ reported recently the use of sperm-hybrid micromotors as cargo-delivery systems for the treatment of gynecological cancers. This highly attractive strategy involves a single sperm cell serving as an active drug (DOX) carrier and as the propulsion force, taking advantage of its swimming capability. A 3D printed four-armed microtube with a nanometric iron layer, called a “tetrapod”, was used to magnetically guide and mechanically release the drug-loaded sperm cell in the desired area (Fig. 3c). The use of sperm cells provides a unique ability to encapsulate hydrophilic drugs owing to their crystalline nuclei, which protect drugs from degradation by the immune response. In addition, drug transfer to the target HeLa cells and spheroids was also improved as a consequence of their somatic cell-fusion ability. Drug delivery occurs when the tubular microstructures bend upon pushing against a tumor spheroid and the sperm squeezes through the cancer cells and fuses with the cell membrane, thus minimizing toxic effects and unwanted drug accumulation in healthy tissues. This actuation mechanism makes these bio-hybrid systems unique and biocompatible vehicles for precise cargo delivery in biomedical applications.

Martel’s group reported the transport of SN-38 drug-loaded nanoliposomes into tumor hypoxic regions using magneto-aerotactic motor-like bacteria, *Magnetococcus marinus* strain MC-1 (Fig. 3d).^48^ In their natural environment, each of the MC-1 cells contains a chain of magnetic iron oxide nanocrystals which allows them to swim along local magnetic field lines. A superior penetration depth in colorectal xenograft tumors was demonstrated compared to passive agents, demonstrating that the swarming behavior of magneto-aerotactic microorganisms can significantly improve the delivery efficiency of drug nanocarriers.

Peng *et al.* ^18^ reported the use of nanosized soft polymer stomatocytes functionalized with PtNPs, used as the engine encapsulated in the cavity of stomatocytes with DOX as the cargo and with chemotactic behavior towards H_2_O_2_ gradients (created either chemically or by excreting neutrophils), as drug carrier systems. The same group recently reported the use of self-assembled biodegradable PtNP-loaded stomatocyte nanomotors, containing poly(ethylene glycol) (PEG), a semicrystalline poly(ε-caprolactone) (PCL) and a glassy polymer (polystyrene, PS) for anticancer drug delivery.^19^ The uptake by HeLa cancer cells and fast DOX drug release of these PEG-*b*-PCL and PEG-*b*-PS-hybrid based stomatocyte nanomotors was demonstrated during degradation under acidic conditions.

Hoop *et al.* ^24^ proposed smart multifunctional tubular nanomachines incorporating a stimuli-responsive building block for targeted drug delivery. The nanomachines consisted of a magnetic Ni nanotube for wireless magnetic propulsion, coated on the outside with Au to allow functionalization with fluorescently tagged thiol-ssDNA. The inner nanotube cavity contained a pH-responsive chitosan hydrogel to carry the drug and selectively release it only in acidic environments. Methylene blue and human epithelial breast cancer cells (MDA-MB-231) were used as model drug and target cells to demonstrate the concept.

Schmidt’s group^25^ introduced for the first time the term medibots, referring to dual-action biogenic hybrid micromotors with functionality both for cell microdrilling and site-directed drug release. These hybrid micromotors are plant-extracted calcified porous microneedles (40–60 μm long), coated *via* e-beam deposition with a magnetic Fe–Ti layer to facilitate cellular drilling by external magnetic actuation. The loading of the calcified biotubes with the anticancer drug camptothecin enabled specific drug release in the acidic environment of HeLa cancer cells.

Chen *et al.* recently reported a hybrid magnetoelectric nanomotor for on-site magnetically-triggered anticancer drug release in breast cancer cells (MDA-MB-231).^64^ In this case, the chemotherapeutic drug paclitaxel was adsorbed onto the polydopamine-modified nanomotor surface and was efficiently released upon the application of alternating magnetic fields due to the magnetoelectric effect.

### Nano- and magnetic particle delivery at the cellular level using nano/microvehicles

Wang’s group reported the design of acoustically propelled shell-shaped nanomotors for the “on-the-move” capture and transport of multiple magnetic cargoes (3.3 μm magnetic beads), and their internalization and propulsion inside live MCF-7 cancer cells.^28^

**Fig. 4:**
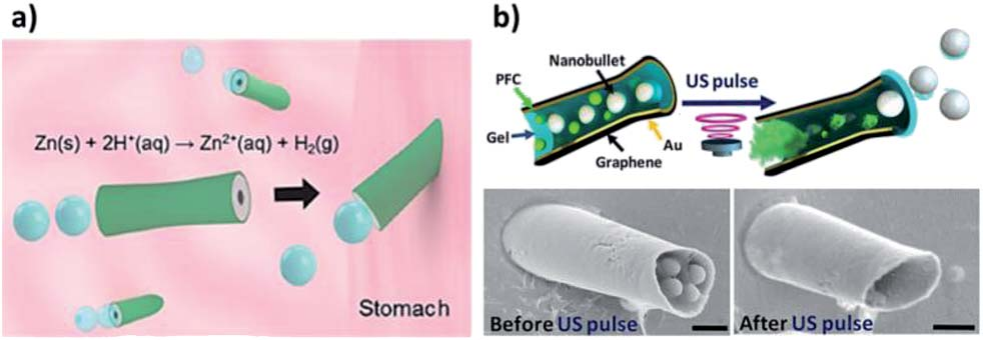
(a) *In vivo* propulsion and tissue penetration of PEDOT/Zn micromotors in a mouse’s stomach. (b) Microcannons for the propulsion of US-triggered nanobullets into diseased tissues. SEM images showing the nanobullet-loaded microcannons before (left) and after (right) US-triggered firing. Reprinted from ref. 16 (a) and ref. 27 (b) with permission.

The same group also reported the first example of an *in vivo* study of artificial PEDOT/Zn micromotors in a living organism, showing efficient propulsion in the harsh acidic environment of mouse stomachs without additional fuel (Fig. 4a).^45^ In this work, the distribution, retention, cargo delivery (using AuNPs as a model) and acute toxicity profile of these acid-driven propelled motors were evaluated *via* oral administration. It was demonstrated that the body of the motors gradually dissolved in gastric acid, thus autonomously releasing the carried payloads on the mouse stomach wall while self-destructing non-toxically.

US-triggered tubular microbullets are extremely promising for addressing the limited tissue penetration challenges of therapeutic particles. Acoustic droplet vaporization was employed for the propulsion of highly powerful perfluorocarbon-loaded microbullets, offering directed drug delivery into diseased tissues.^67^ A similar technology was reported by Soto *et al.* to develop acoustically-triggered microcannons capable of loading and firing nanobullets (Fig. 4b).^27^ The constructed microcannons could allow the efficient loading and firing of nanoscale cargoes as nanoprojectiles, favoring the direct and deep penetration of therapeutics into tissues. Such technology is expected to be the next generation of efficient nanoscale delivery devices, capable of delivering different drug cocktails and vaccines into identified targets.

### Cell delivery at the cellular level using nano/microvehicles

The Schmidt group presented a novel fertilization method involving the use of artificially motorized sperm cells, where customized metal-coated polymer microhelices served as motors for transporting sperm cells with motion deficiencies to help them to carry out their natural function.^31^ The reported method demonstrated the active capture, transport and efficient delivery of a single live sperm cell to an oocyte cell wall.

### Gene/protein delivery at the cellular level using nano/microvehicles

Gene or protein therapy implies the use of DNA or protein therapeutic substances, which are delivered into a patient’s cells to treat diseases such as inherited disorders and cancers.^23,32^ Fan *et al.* ^63^ demonstrated that electrically propelled protein-functionalized AuNW motors could deliver “on-the-fly” cytokine tumor necrosis factor alpha (TNFα) and activated canonical nuclear factor-kappaB (NF-kB) signaling to single HeLa cancer cells.

ABFs functionalized (f-ABFs) with complexes of cationic lipids and plasmid DNA (lipoplexes) were proposed by Qiu *et al.* ^23^ for *in vitro* wirelessly targeted single-cell gene delivery to human embryonic kidney (HEK 293) cells. The successful *in vitro* transfection of the HEK 293 cells by f-ABFs, steered wirelessly by low-strength rotating magnetic fields, was demonstrated by expressing the encoding Venus protein.

**Fig. 5:**
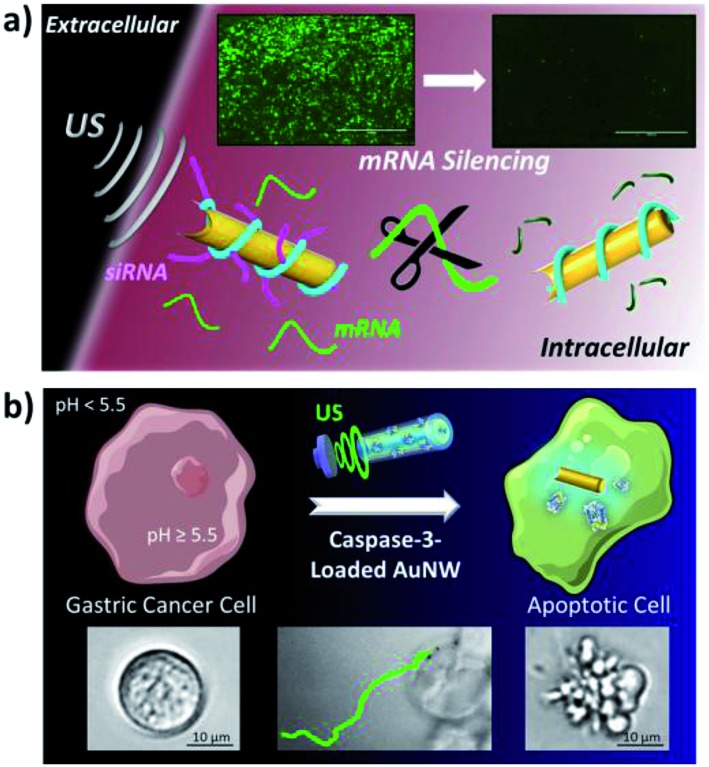
US-propelled AuNWs for intracellular gene (a) and protein (b) delivery. (a) An intracellular GFP gene silencing strategy, through siRNA delivery anchored to a RCA DNA strand wrapped around acoustically propelled AuNWs. (b) Directed apoptosis of a target cell by an efficient cytosolic delivery strategy using active CASP-3 encapsulated within a biocompatible high pH-responsive polymeric coating on US-propelled AuNW motors. Reprinted from ref. 29 (a) and ref. 30 (b) with permission.

The advantages of the rapid internalization and intracellular movement of US-powered nanomotors have also been exploited for intracellular oligonucleotide delivery. Wang’s group described recently an effective intracellular gene silencing strategy, using acoustically-propelled AuNWs wrapped with a Rolling Circle Amplification (RCA) DNA strand, which served to anchor green fluorescent protein (GFP) which was targetedly interfering with the RNA’s (siGFP) payload (Fig. 5a).^29^ The US-propulsion led to the fast internalization and rapid intracellular movement of the AuNWs, and hence to an accelerated siRNA delivery and silencing response. The strategy allowed 94% silencing of the GFP response in two different cell lines (HEK-293-GFP and MCF-7-GFP) after 5 min treatment with the GFP/RCA-wrapped AuNWs. Interestingly, most of the cells remained alive after the nanomotor treatment, even when using a high nanomotor concentration.

The same group has described recently a method based on the use of US-propelled nanomotors coated with a high pH-responsive polymer, for the rapid internalization and cytosolic delivery of functional caspase-3 (CASP-3), with the aim of causing apoptosis in human gastric adenocarcinoma cells (Fig. 5b).^30^ The nanomotor allowed 80% apoptosis of cancer cells within only 5 min, suggesting that such nanovehicles may constitute powerful tools for the cytosolic delivery of active therapeutic proteins. Compared to other apoptosis approaches, the nanomotor strategy resulted in the highest apoptosis efficiency while requiring significantly shorter times and smaller amounts of CASP-3.

It is worth mentioning that the extraordinary capabilities demonstrated by fuel-free nano/micromotors for targeted gene or protein delivery open the door for a wide portfolio of potential applications in the fields of therapy bioengineering and cell biology in various *in vitro* and *in vivo* settings. Such intracellular delivery can thus sharply increase the efficiency of various treatment protocols.

## Key challenges, envisioned opportunities and future perspectives

The discussed examples demonstrate the broad range of applications performed at the cellular level using nano/micromotors. They constitute clear proof of the remarkable progress made in the design and construction of these tiny machines to integrate self-driven, navigation and biosensing capabilities, and perform multitasking, including drug loading, targeted transportation and autonomous or remotely-controlled release in complex environments. These capabilities make them extremely promising vehicles to be employed for biosensing and drug release applications at the cellular level. The reports described so far demonstrate that different types of nano/micromotor can be used as suitable engines for operation in cellular environments. Moreover, at the cellular level, the biosensing applications of nano/micromotors have been much less exploited than those involving cargo delivery (mainly of chemotherapeutic drugs). Such new nanovehicles thus offer considerable promise for the monitoring of complex processes that occur within cells and for modulating such cellular processes and increasing the efficiency of various treatment protocols. While several novel intracellular applications of nanovehicles have been covered in this article, the ability to sense cellular processes and deliver payloads within the cell is still at a very early stage. Future advances in nano/micromotors will be critical to biomedical applications that rely on sensing, delivery and targeting in intracellular space.

The studies discussed above show that these smart devices, self-propelled or externally powered by catalytic, magnetic or acoustic sources, offer a myriad of mechanical movements and broad design versatility in terms of building block materials (silicon-based materials, polymers or noble and other metals), size (ranging from the nanoscale to the microscale) and shape (wires, spheres and tubes/rockets). Since each specific shape and building block material demands a given fabrication technique, determining important functioning properties apart from the vehicle motion mechanism, the design and preparation of motors devoted to a specific application requires the integration of several crucial factors. On the positive side, the large number of available possibilities to tailor the behavior and properties of the constructed engines considerably increases their potential applications.

The developed smart vehicles demonstrated outstanding capabilities in terms of speed and force, drug loading and towing capacity (easily tunable through fabrication and by changing the fuel concentration, magnetic and UV field strengths and temperature), biocompatibility and biodegradability, wireless transport to specific hard-to-reach areas and feasibility to carry a wide variety of cargoes (drugs, genes, enzymes, cells and other relevant chemicals) and release them using biocompatible mechanisms (pH responsive, NIR irradiation and magnetic actuation). Importantly, the biosensing and delivery missions undertaken have been performed both at extra- and intra-cellular levels without compromising the viability of the target cells. While a wide variety have been applied in extracellular space, the intracellular applications have been demonstrated primarily using acoustic propelled nano/micromotors.

However, it is important to mention that most of these remarkable capabilities of nano/micromotors have been demonstrated only *in vitro*, and potential difficulties might be encountered for *in vivo* applications. In addition to the limitation of using fuel-free or biocompatible fuel nanomotors, there are some serious challenges to face to achieve *in vivo* applications. The smart vehicles must be able to evade the immune system of the host organism and, in the specific case of liposome-based systems, destruction by the reticuloendothelial system. Drug delivery should also overcome the high interstitial pressure in late stage tumors, the endosomal escape challenge and the natural physiological barriers such as the highly acidic gastric environment. Moreover, for *in vivo* applications, the nano/micromotor must be readily degraded into non-toxic compounds without interference from outside through passive or built-in self-destruction mechanisms activated after finishing their mission.^68^ Novel smart materials, including biological, responsive or soft materials, are therefore highly desired to provide triggered autonomous actuation and multifunctionality while avoiding irreversible malfunctions in complex physiologically relevant body systems.

Additional efforts should also be devoted towards developing functional geometries and exploring new synthesis methods for the easy, large scale, high quality, cost-efficient and environmentally friendly fabrication of nano/micromotors. The identification of new energy sources for enhancing tissue penetration and achieving prolonged, biocompatible and fully autonomous operation in complex biological media such as blood (with large drag forces associated with the high viscosity of the blood cells) should also be pursued. Micromotors powered by bio-friendly fuels also require additional improvements for their practical implementation, due to the relatively short lifetimes of those using active material propellants (*e.g.*, Mg, Zn, Al and CaCO_3_) and the limited power and stability of enzyme-based ones. In this sense, coupling synthetic nanomachines with natural biological materials can minimize these problems, apart from the desired immune evasion and biofouling occurring in complex biological fluids, leading to their enhanced mobility and extended lifetime. The future biomedical operation of nano/micromachines will require individual control of the nano/micromotors wirelessly, for example by the integration of nanoscale control systems and the development and coupling with high-resolution imaging and feedback control systems for the simultaneous localization and mapping of multiple nano/micromotors in the human body.

It is worth noting that despite the long and challenging road ahead, some important steps have been already achieved to address these important issues. The stabilization of the substrates and application of appropriate coatings or preconditioning to the liposomes/cargoes have been employed in the fabrication processes of nano/micromotors to potentially address the endosome escape challenge. Moreover, highly hemo-compatible nano/microvehicles, with a negligible influence from red blood cells on their motion, have already been developed, and more biocompatible nano/micromotors have been proposed involving coatings with different biocompatible materials, such as red blood cell membranes. Most interestingly although still scarcely explored, some authors have already reported the appropriate operation and behavior of these smart systems in *in vivo* environments.^45^ These pioneering applications have demonstrated the capabilities of nano/micromotors for safely and rapidly yet transiently neutralizing gastric acid, and simultaneously releasing their payload without affecting the stomach function or causing adverse effects in living animals. This type of work represents an important step forward for clinical translation and paves the way for many other breakthrough *in vivo* applications. The limited tissue penetration has been addressed using US-powered tubular microbullets, in which the ‘bullet-like’ propulsion allows the vehicle to reach different depths of the tissue to perform precise nanosurgery, as well as enabling it to carry large payloads. Indeed, the latest applications in this field have even demonstrated the use of nano/micromotors as efficient vehicles for the direct cytosolic delivery of active therapeutic proteins after internalization in intact target cells. While progress has been made in this direction, effective intracellular delivery to the cytosol remains a challenge. Future efforts could lead to the targeting of specific cytoplasmic organelles, such as the mitochondria, through guided movement within the intracellular space. It is also worth mentioning that, although only cargo delivery applications have been considered in this review, the nearly limitless possibilities demonstrated by nano/microvehicles to deliver a wide variety of payloads with diverse biomedical functions also open up a wide range of other opportunities for therapy, diagnostics and imaging.

Advances in this field will likely come from the integration of the tremendous progress made in nano/micromotor research during the last few years with a variety of exciting nano/micromotor-based strategies for the biosensing, transport and release of therapeutic agents at the cellular level, with advances in materials science, nanotechnology and molecular biology. This marriage will be essential to overcome the remaining challenges and promote the wide implementation of these smart, tiny multitasking vehicles for *in vivo* biosensing and cargo delivery. Furthermore, to fully realize the abilities of nano/micromotors in medicine, a closer collaboration between scientists working in the development of nano/micromachines and medical researchers will be required for designing and constructing devices able to meet the demands and needs of the medical community. Overcoming these challenges to further increase the power and functionality of nano/micromotors, together with finding effective paths to take advantage of the emerging novelties in this exciting field toward the most relevant practical and commercial applications demanded by clinical experts, will be compulsory to ensure their utilization in diagnostics and therapy, which is still in its infancy. Therefore, substantial efforts and new innovations are still required to realize the full potential of these tiny motors in a new generation of nano/micromotors. Ideally, they should be fabricated using cutting-edge techniques, be able to mimic the natural intelligence of their biological counterparts, exhibit improved, adaptable and sustainable operation in biological media, have a deformable structure, be able to be precisely controlled, and have the ability to act both individually and collectively with synchronized coordination and self-adaptive and self-replicating capabilities. Nevertheless, the remarkable performance already demonstrated by nano/micromotors to design problem-oriented medical devices for specific sensing or delivery functions undoubtedly represents a tremendous asset in diagnostics and therapeutics. Therefore, the accelerated translation of nano/micromotor research into practical clinical use nowadays seems more reality than science fiction.

## Conflicts of interest

There are no conflicts to declare.

## References

[cit1] Wang J., Nanomachines: fundamentals and applications, Wiley-VCH, 2013.

[cit2] Moreno-Guzmán M., Jodra A., López M.-Á., Escarpa A. (2015). Anal. Chem..

[cit3] Wang J., Gao W. (2012). ACS Nano.

[cit4] Li J., Esteban-Fernández de Ávila B., Gao W., Zhang L., Wang J. (2017). Science Robotics.

[cit5] Sánchez S., Soler L., Katuri J. (2014). Angew. Chem., Int. Ed..

[cit6] Sokolov I. L., Cherkasov V. R., Tregubov A. A., Buiucli S. R., Nikitin M. P. (2017). Biochim. Biophys. Acta.

[cit7] Mou F., Li Y., Chen C., Yin W. Y., Ma H., Guan J. (2015). Small.

[cit8] Rao K. J., Li F., Meng L., Zheng H., Cai F., Wang W. (2015). Small.

[cit9] Xu T., Gao W., Xu L.-P., Zhang X., Wang S. (2017). Adv. Mater..

[cit10] Solovev A. A., Mei Y., Bermudez Urena E., Huang G., Schmidt O. G. (2009). Small.

[cit11] Balasubramanian S., Kagan D., Jack Hu C.-M., Campuzano S., Lobo-Castañón M. J., Lim N., Kang D. Y., Zimmerman M., Zhang L., Wang J. (2011). Angew. Chem., Int. Ed..

[cit12] Campuzano S., Orozco J., Kagan D., Guix M., Gao W., Sattayasamitsathit S., Claussen J. C., Merkoçi A., Wang J. (2012). Nano Lett..

[cit13] Wu Y., Lin X., Wu Z., Möhwald H., He Q. (2014). ACS Appl. Mater. Interfaces.

[cit14] Xuan M., Shao J., Lin X., Dai L., He Q. (2014). ChemPhysChem.

[cit15] Wu Z., Lin X., Zou X., Sun J., He Q. (2015). ACS Appl. Mater. Interfaces.

[cit16] Gao W., Dong R., Thamphiwatana S., Li J., Gao W., Zhang L., Wang J. (2015). ACS Nano.

[cit17] Guix M., Meyer A. K., Koch B., Schmidt O. G. (2016). Sci. Rep..

[cit18] Peng F., Tu Y., van Hest J. C. M., Wilson D. A. (2015). Angew. Chem., Int. Ed..

[cit19] Tu Y., Peng F., André A. A. M., Men Y., Srinivas M., Wilson D. A. (2017). ACS Nano.

[cit20] Ghosh A., Fischer P. (2009). Nano Lett..

[cit21] Gao W., Kagan D., Pak O. S., Clawson C., Campuzano S., Chuluun-Erdene E., Shipton E., Fullerton E. E., Zhang L., Lauga E., Wang J. (2012). Small.

[cit22] Mhanna R., Qiu F., Zhang L., Ding Y., Sugihara K., Zenobi-Wong M., Nelson B. J. (2014). Small.

[cit23] Qiu F., Fujita S., Mhanna R., Zhang L., Simona B. R., Nelson B. J. (2015). Adv. Funct. Mater..

[cit24] Hoop M., Mushtaq F., Hurter C., Chen X.-Z., Nelson B. J., Pané S. (2016). Nanoscale.

[cit25] Srivastava S. K., Medina-Sánchez M., Koch B., Schmidt O. G. (2016). Adv. Mater..

[cit26] Garcia-Gradilla V., Sattayasamitsathit S., Soto F., Kuralay F., Yardımcı C., Wiitala D., Galarnyk M., Wang J. (2014). Small.

[cit27] Soto F., Martin A., Ibsen S., Vaidyanathan M., Garcia-Gradilla V., Levin Y., Escarpa A., Esener S. C., Wang J. (2016). ACS Nano.

[cit28] Soto F., Wagner G. L., Garcia-Gradilla V., Gillespie K. T., Lakshmipathy D. R., Karshalev E., Angell C., Chen Y., Wang J. (2016). Nanoscale.

[cit29] Esteban-Fernández de Ávila B., Angell C., Soto F., Lopez-Ramirez M. A., Báez D. F., Xie S., Wang J., Chen Y. (2016). ACS Nano.

[cit30] Esteban-Fernández de Ávila B., Ramírez-Herrera D. E., Campuzano S., Angsantikul P., Zhang L., Wang J. (2017). ACS Nano.

[cit31] Medina-Sánchez M., Schwarz L., Meyer A. K., Hebenstreit F., Schmidt O. G. (2016). Nano Lett..

[cit32] Xu H., Medina-Sánchez M., Magdanz V., Schwarz L., Hebenstreit F. and Schmidt O. G., Sperm-hybrid micromotor for drug delivery in the female reproductive tract, Physics Medical Physics, arXiv:1703.08510.

[cit33] Li J., Rozen I., Wang J. (2016). ACS Nano.

[cit34] Wang J., Manesh K. M. (2010). Small.

[cit35] Zhang H., Duan W., Lu M., Zhao X., Shklyaev S., Liu L., Huang T. J., Sen A. (2014). ACS Nano.

[cit36] Sengupta S., Patra D., Ortiz-Rivera I., Agrawal A., Shklyaev S., Dey K. K., Cordova-Figueroa U., Mallouk T. E., Sen A. (2014). Nat. Chem..

[cit37] Ma X., Jannasch A., Albrecht U. R., Hahn K., Miguel-Lopez A., Schaffer E., Sanchez S. (2015). Nano Lett..

[cit38] Ma X., Wang X., Hahn K., Sanchez S. (2016). ACS Nano.

[cit39] Gao W., Pei A., Wang J. (2012). ACS Nano.

[cit40] Gao W., Feng X., Pei A., Gu Y., Li J., Wang J. (2013). Nanoscale.

[cit41] Mou F., Chen C., Ma H., Yin Y., Wu Q., Guan J. (2013). Angew. Chem., Int. Ed..

[cit42] Li J., Singh V. V., Sattayasamitsathit S., Orozco J., Kaufmann K., Dong R., Gao W., Jurado-Sanchez B., Fedorak Y., Wang J. (2014). ACS Nano.

[cit43] Wu Z., Li J., Esteban-Fernández de Ávila B., Li T., Gao W., He Q., Zhang L., Wang J. (2015). Adv. Funct. Mater..

[cit44] Peng F., Tu Y., Wilson D. A. (2017). Chem. Soc. Rev..

[cit45] Li J., Thamphiwatana S., Liu W., Esteban-Fernández de Ávila B., Angsantikul P., Sandraz E., Wang J., Xu T., Soto F., Ramez V., Wang X., Gao W., Zhang L., Wang J. (2016). ACS Nano.

[cit46] Li J., Angsantikul P., Liu W., Esteban-Fernández de Ávila B., Thamphiwatana S., Xu M., Sandraz E., Wang X., Delezuk J., Gao W., Zhang L., Wang J. (2017). Angew. Chem., Int. Ed..

[cit47] Qiu F., Mhanna R., Zhang L., Ding Y., Fujita S., Nelson B. J. (2014). Sens. Actuators, B.

[cit48] Felfoul O., Mohammadi M., Taherkhani S., de Lanauze D., Zhong Xu Y., Loghin D., Essa S., Jancik S., Houle D., Lafleur M., Gaboury L., Tabrizian M., Kaou N., Atkin M., Vuong T., Beauchemin G. N., Radzioch D., Martel S. (2016). Nat. Nanotechnol..

[cit49] Wang W., Li S., Mair L., Ahmed S., Huang T. J., Mallouk T. E. (2014). Angew. Chem., Int. Ed..

[cit50] Magdanz V., Sanchez S., Schmidt O. G. (2013). Adv. Mater..

[cit51] Chałupniak A., Morales-Narváez E., Merkoçi A. (2015). Adv. Drug Delivery Rev..

[cit52] Gao W., Sattayasamitsathit S., Orozco J., Wang J. (2011). J. Am. Chem. Soc..

[cit53] Campuzano S., Kagan D., Orozco J., Wang J. (2011). Analyst.

[cit54] Wang J. (2016). Biosens. Bioelectron..

[cit55] Kagan D., Campuzano S., Balasubramanian S., Kuralay F., Flechsig G.-U., Wang J. (2011). Nano Lett..

[cit56] Babu A., Templeton A. K., Munshi A., Ramesh R. (2014). AAPS PharmSciTech.

[cit57] Davis M. E., Chen Z., Shin D. M. (2008). Nat. Rev. Drug Discovery.

[cit58] Gao W., Wang J. (2015). Nanoscale.

[cit59] Sattayasamitsathit S., Kou H., Gao W., Thavarajah W., Kaufmann K., Zhang L., Wang J. (2014). Small.

[cit60] Guix M., Mayorga-Martinez C. C., Merkoçi A. (2014). Chem. Rev..

[cit61] Kuralay F., Sattayasamitsathit S., Gao W., Uygun A., Katzenberg A., Wang J. (2012). J. Am. Chem. Soc..

[cit62] Esteban-Fernández de Avila B., Martín A., Soto F., López-Ramirez M. A., Campuzano S., Vásquez-Machado G. M., Gao W., Zhang L., Wang J. (2015). ACS Nano.

[cit63] Fan D., Yin Z., Cheong R., Zhu F. Q., Cammarata R. C., Chien C. L., Levchenko A. (2010). Nat. Nanotechnol..

[cit64] Chen X.-Z., Hoop M., Shamsudhin N., Huang T., Özkale B., Li Q., Siringil E., Mushtaq F., Di Tizio L., Nelson B. J., Pané S. (2017). Adv. Mater..

[cit65] Kagan D., Laocharoensuk R., Zimmerman M., Clawson C., Balasubramanian S., Kang D., Bishop D., Sattayasamitsathit S., Zhang L., Wang J. (2010). Small.

[cit66] Wu Z., Wu Y., He W., Lin X., Sun J., He Q. (2013). Angew. Chem., Int. Ed..

[cit67] Kagan D., Benchimol M. J., Claussen J. C., Chuluun-Erdene E., Esener S., Wang J. (2012). Angew. Chem., Int. Ed..

[cit68] Chen C., Karshalev E., Li J., Soto F., Castillo R., Campos I., Mou F., Guan J., Wang J. (2016). ACS Nano.

